# A novel CD74-ROS1 gene fusion in a patient with inflammatory breast cancer: a case report

**DOI:** 10.1186/s13256-021-02876-5

**Published:** 2021-05-30

**Authors:** Huiyu Hu, Nianhua Ding, Haiyan Zhou, Shouman Wang, Lili Tang, Zhi Xiao

**Affiliations:** 1 Department of General Surgery, Xiangya Hospital, Central South University, Xiangya Road 87#, Changsha, Hunan People’s Republic of China; 2 Clinical Research Center for Breast Cancer Control and Prevention in Hunan Province, Changsha, China; 3 Department of clinical laboratory, The First Hospital of Changsha, Changsha, China; 4 Department of Pathology, Xiangya Hospital, Central South University, Changsha, China

**Keywords:** Inflammatory breast neoplasms, Gene fusion, Case report

## Abstract

**Background:**

CD74-ROS1 fusion genes have been detected in non-small cell lung carcinomas (NSCLC), but not in inflammatory breast cancer.

**Case presentation:**

Herein, we report a CD74-ROS1 fusion gene identified in a 64-year-old Chinese woman with inflammatory breast cancer (IBC). The patient initially presented with a rapidly growing mass in the left breast with diffuse erythema developing over a period of 2 months. Diagnosis of invasive breast carcinoma was made by core needle biopsy. Positron emission tomography-computed tomography (PET/CT) demonstrated multiple organ metastases. Genomic DNA was extracted from tumor tissue and analyzed using next-generation sequencing (NGS). The CD74-ROS1 fusion gene was detected in the genomic DNA. The patient refused crizotinib treatment, and could not tolerate the side effects of palliative chemotherapy. Unfortunately, the patient died 4 months after diagnosis.

**Conclusion:**

We report the case of a CD74-ROS1 fusion gene in a patient with IBC. This may reveal, for the first time, a possible association between CD74-ROS1 gene fusion and rapid progression of inflammatory breast cancer. Multigene panel testing can be performed when rapidly progressive breast cancer occurs and could reveal potential therapeutic strategies.

## Background

Inflammatory breast cancer (IBC) is a rare and aggressive disease that accounts for ~ 2.5% of all invasive breast cancer and is characterized by rapid progression and worse prognosis [1]. The overall survival of IBC patients is shorter than that of non-IBC patients; several studies show 5-year and 15-year overall survival of approximately 40% and 20%, respectively [1–3]. The identification of oncogenic drivers and subsequent targeting of these proteins could be a promising treatment strategy for IBC. Next-generation sequencing (NGS) technology provides a highly efficient method to obtain vast genomic information which could be useful for treatment selection. In this study, we report a CD74-ROS1 fusion gene detected by NGS in a patient with IBC. Based on a literature search, chromosomal rearrangements involving the ROS1 gene were first described in non-small cell lung carcinoma (NSCLC), and CD74-ROS1 fusion transcripts were also detected in NSCLC [3, 4]. A CD74-ROS1 fusion gene in breast cancer has never been reported in the literature.

## Case presentation

**Fig. 1.**
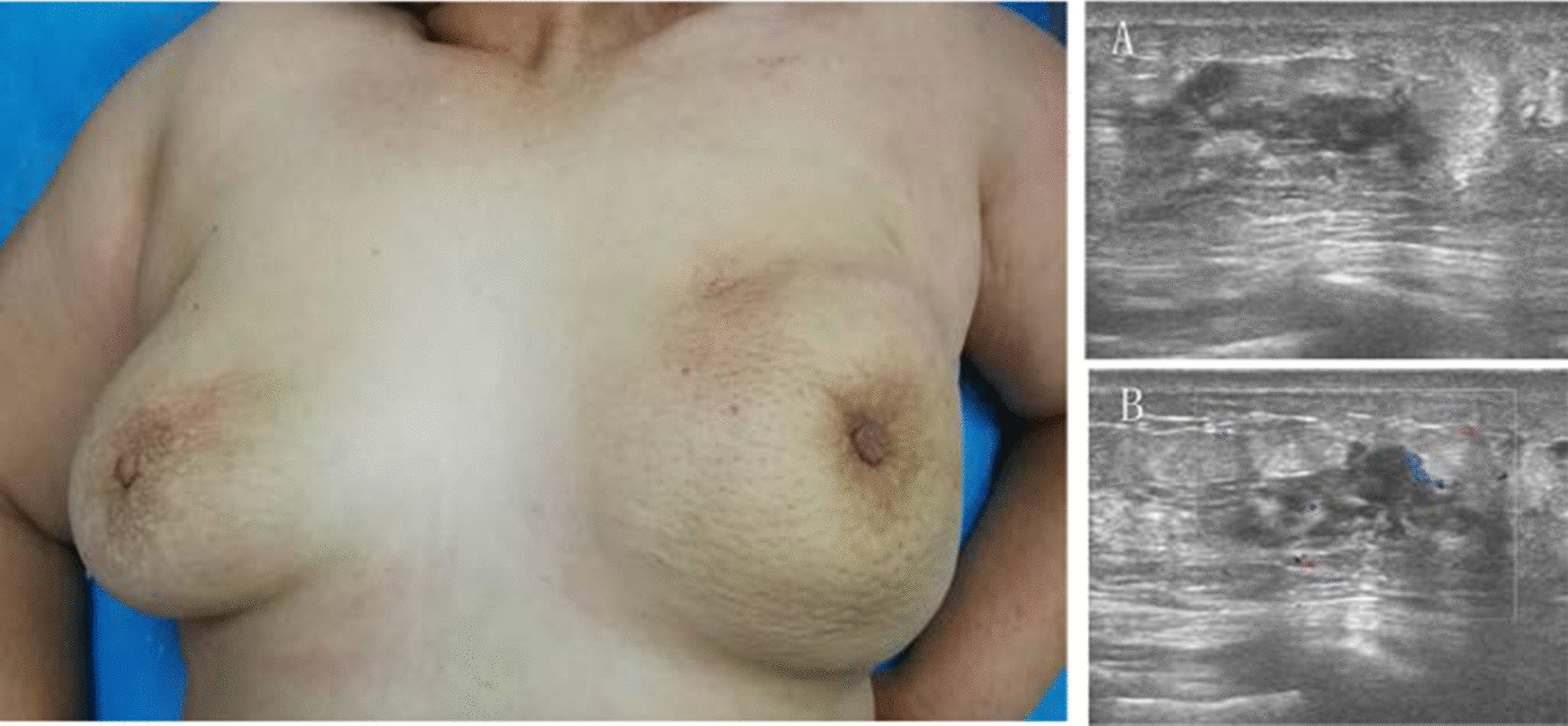
Gross finding and ultrasound (US) examination. Diffuse erythema, skin ridging and peau d’orange appearance of the left breast skin. **a** US showed a hypoechoic area with irregular shape and unclear border. **b** Color Doppler US showed increased blood flow within the lesion.

**Fig. 2.**
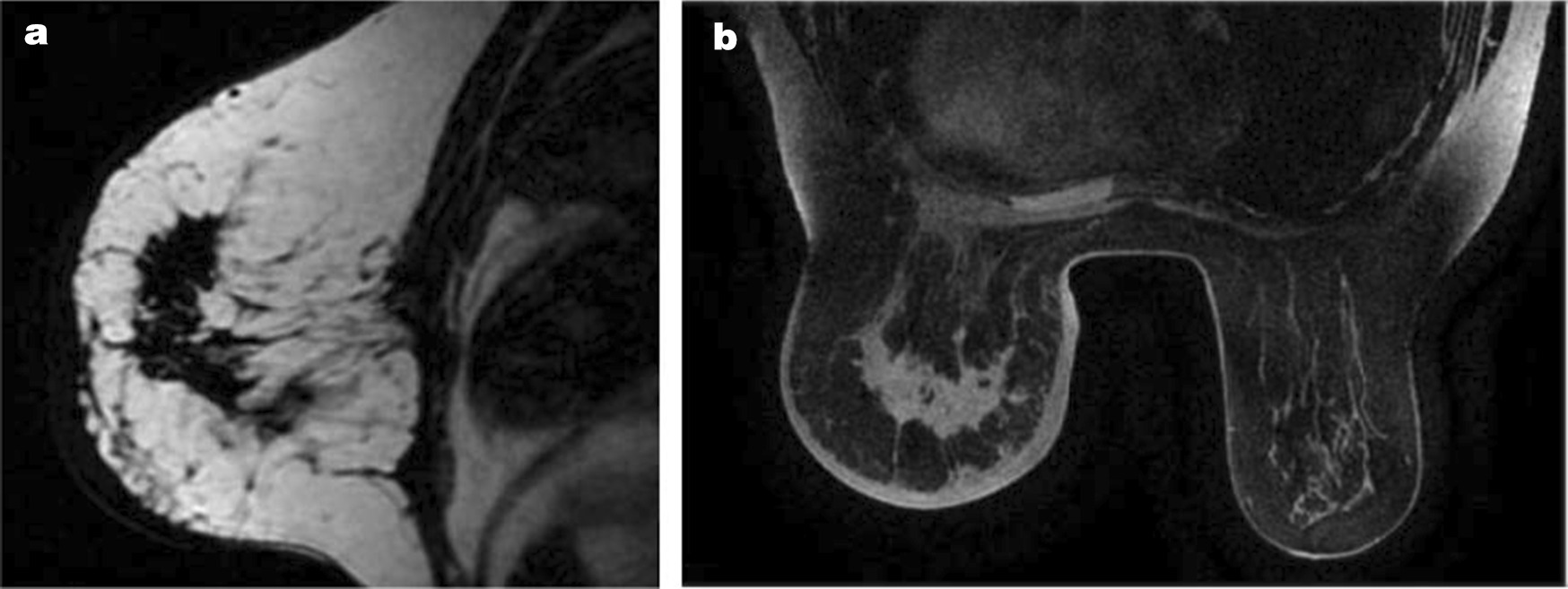
Magnetic resonance imaging (MRI). MRI showed the heterogeneously distributed lesion in an area of high signal intensity in the left breast on T1-weighted images. The breast mass was rapidly enhanced following the injection of gadolinium diethylenetriamine-pentaacetic acid at an early phase (2 minutes after agent injection).

**Fig. 3.**
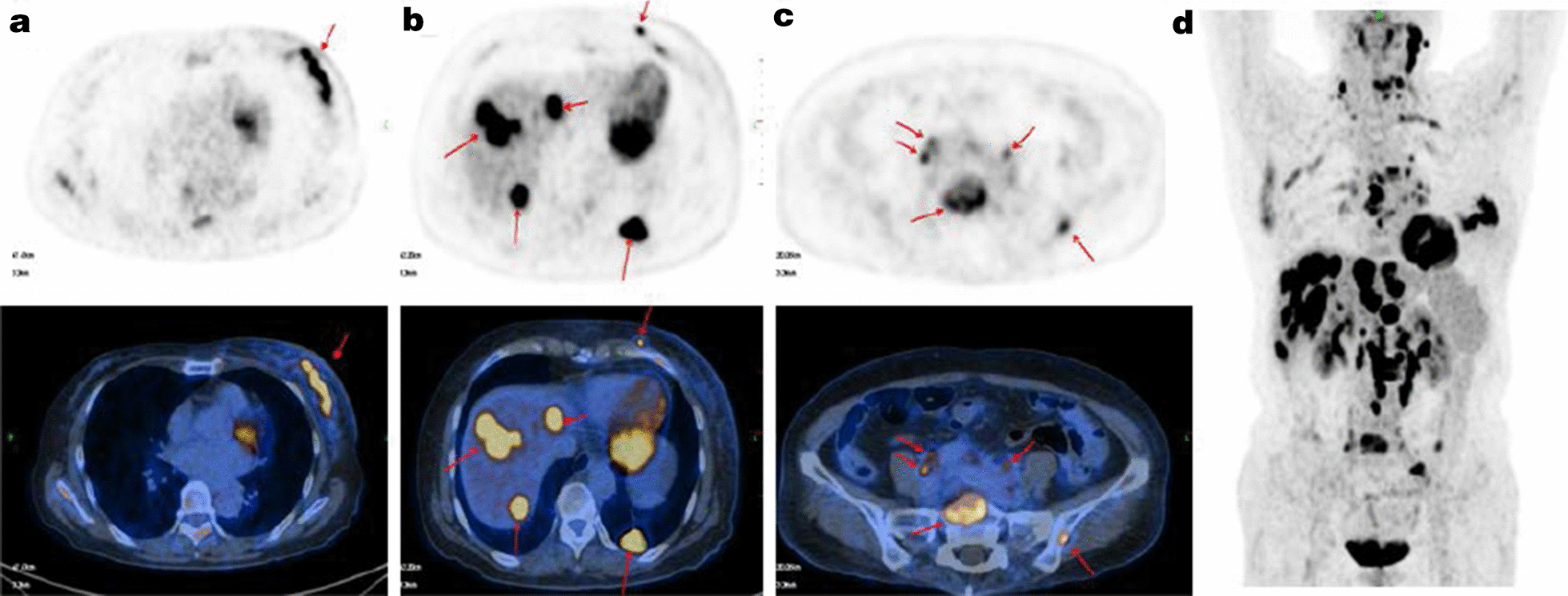
Positron emission tomography-computed tomography (PET/CT) imaging. PET/CT indicated increased ^18^F-fluorodeoxyglucose uptake in the left breast (**a**), in liver (**b**) and bilateral iliac blood vessels lymph nodes and bones (**c**). The arrow indicated the point that had increased ^18^F-fluorodeoxyglucose uptake. Showed the 310 increased ^18^F-fluorodeoxyglucose uptake in the whole body (**d**).

**Fig. 4.**
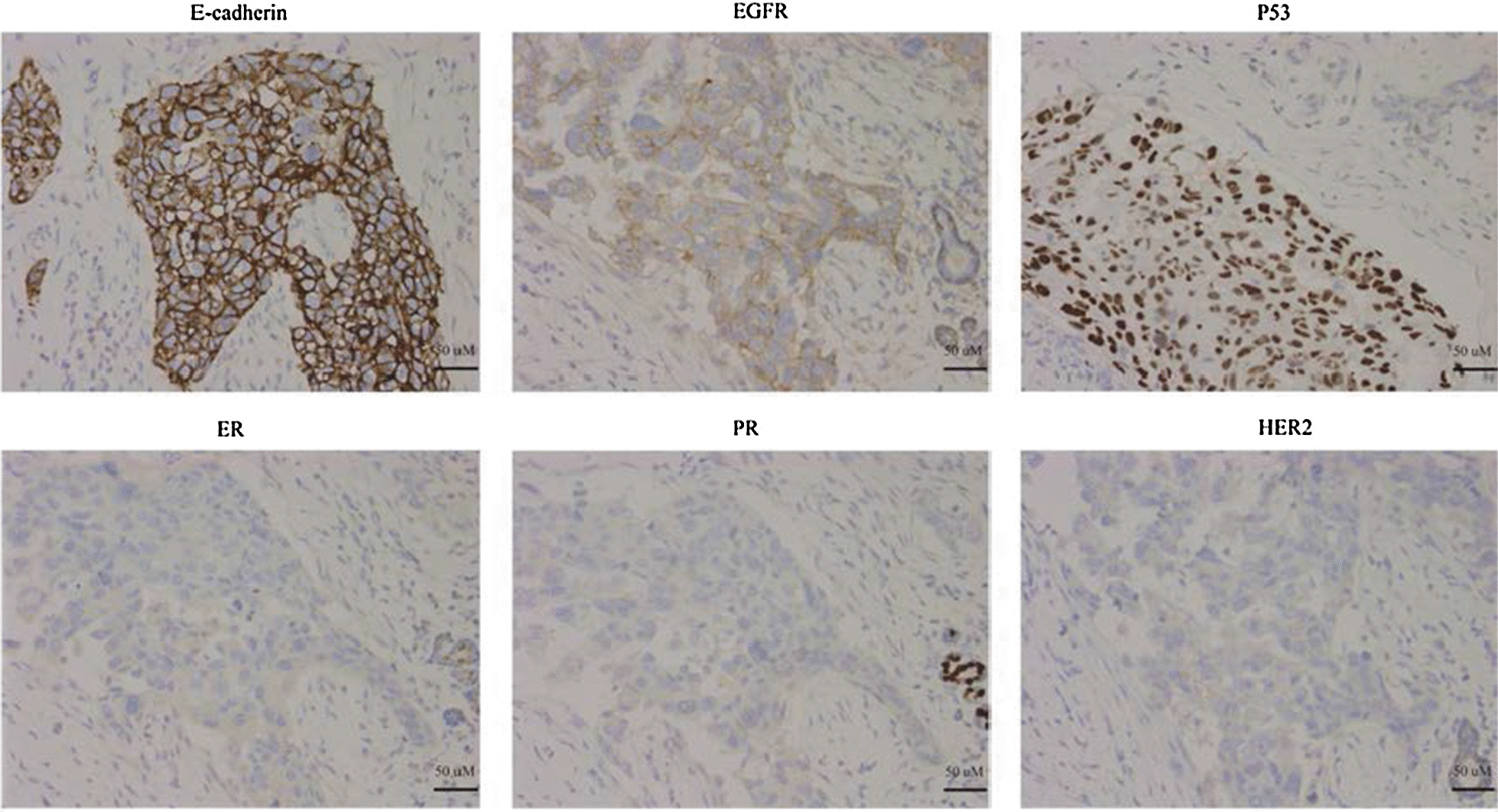
Immunohistochemical staining imaging. The breast carcinoma was immunohistochemically positive for E-cadherin, epidermal growth factor receptor (EGFR) and P53, and negative for estrogen receptor, progesterone receptor and HER2.

**Fig. 5.**
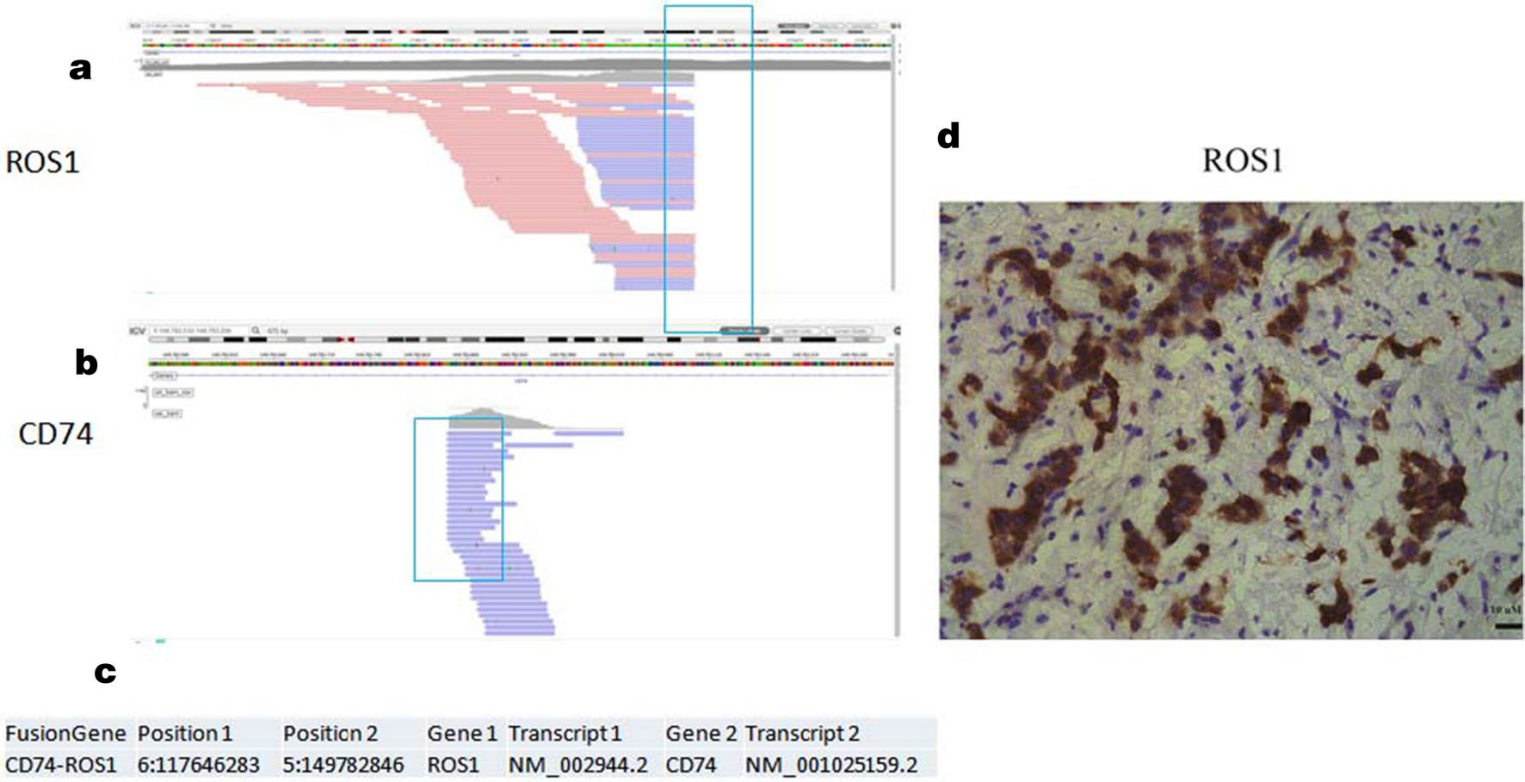
CD74-ROS1 fusion gene examination. **a** The reads map sequenced on ROS1. Each line represents a read. Pink or purple color represents sequenced reads in different directions. **b** The reads map of CD74. The neat truncation position of the frames indicates the breakpoint of the fusion of two genes. **c** The table shows the fusion position of CD74-ROS1. **d** The immunohistochemistry image for the ROS1 protein. The overexpression of the ROS1 protein was detected specifically in the cytoplasm of cancerous cells.

A 64-year-old Chinese woman presented to the clinic with a painless mass on the left breast. The mass had been present for approximately 1 year, with relatively slow progression, and had only rapidly increased in size in the past 2 months. She had no remarkable family history. A physical examination revealed diffuse erythema, skin ridging and peau d’orange appearance of the skin (Fig. 1). Breast mass size was 7.0 cm×6.5 cm. There were multiple enlarged and fused lymph nodes in the axilla and supraclavicular region. Ultrasound showed a hypoechoic area with irregular shape and unclear border (Fig. 1). Internally, the hypoechoic area was asymmetrical, and a strong increase in blood flow was detected within the lesion. Ultrasound also showed enlarged lymph nodes in the axilla and supraclavicular region. Dynamic contrast-enhanced magnetic resonance imaging (MRI) showed a heterogeneously distributed lesion of high signal intensity in the left breast on T1-weighted images. The breast mass was rapidly enhanced following the injection of gadolinium diethylenetriamine-pentaacetic acid (Fig. 2). Positron emission tomography-computed tomography (PET/CT) indicated increased ^18^F-fluorodeoxyglucose uptake in the left breast, in the nodes of the chest wall and the nodes of the left posterior basal lung. PET/CT also demonstrated additional uptake in the internal mammary, mediastinum (4R, 4L, 5 and 7 groups) and hilum of the lung (10 and 11 groups), and bilateral cervical, retroperitoneal and bilateral iliac blood vessel lymph nodes. Increased uptake in the liver was observed as well (Fig. 3). Core needle aspiration was performed for the diagnosis of invasive ductal carcinoma of the left breast in July 2017. Diagnosis of breast carcinoma was made by both hematoxylin and eosin (HE) staining and immunostaining. This breast carcinoma was immunohistochemically negative for estrogen receptor (ER), progesterone receptor (PR) and human epidermal growth factor receptor 2 (HER2). The carcinoma was immunohistochemically positive for P53, E-cadherin and epidermal growth factor receptor (EGFR) (Fig. 4). Based on these pathological results, combined with the clinical characteristics, this patient was diagnosed with IBC (cT4N3M1). The patient was enrolled in a clinical study analyzing IBC genomic mutation status using NGS. Following verbal and written informed consent, genomic DNA was extracted from tumor tissue. Tumor tissue DNA was sequenced by target region capture sequencing using a previously described NGS technique [5]. TP53BP1 and ARID1A nonsense mutations and a CD74-ROS1 fusion gene were detected in the genomic DNA. The CD74-ROS1 fusion gene combined an exon 7 of CD74 and an exon 34 of ROS1, and the ROS1 protein was overexpressed, driven by this fusion oncogene in breast tumor tissues (Fig. 5).

Chemotherapy (paclitaxel, 120mg) was given every week for a period of 3 weeks. Oral administration of capecitabine tablets (1.5 g twice daily, days 1–14 of a 21-day cycle) was given for three cycles due to unacceptable anorexia with paclitaxel. The patient refused crizotinib treatment for economic reasons and was confirmed dead in November 2017.

## Discussion and conclusion

A CD74-ROS1 fusion gene, which has yet to be reported in a breast cancer patient, was detected in a 64-year-old Chinese woman diagnosed with IBC. This patient did not have any family history of cancer. The detected gene fusion occurred between exon 7 of CD74 and exon 34 of ROS1. ROS1 proteins were overexpressed in tumor tissue. The *ROS1* gene, located on chromosome 6q22.1, encodes a receptor tyrosine kinase (RTK), and is related to cell differentiation located in specific organs. In a mouse model, transient ROS1 expression during development in the kidney, intestine and lung coincided with major morphogenetic and differentiation events in these organs [6]. Furthermore, ROS1 transcripts were directly involved in inductive interactions between epithelial and mesenchymal cells in kidney and intestine development [6, 7]. In mice, ROS1 deficiency induced inappropriate regionalization and terminal differentiation of epithelial cells in the epididymis, whereas female ROS1-deficient mice developed normally with no affects on fertility [8]. Human ligand of ROS1 has not yet been identified, and the function of ROS1 needs further investigation [9].

A literature review revealed only one abstract reporting the occurrence of ROS1 fusion in breast cancer [10], and showed the alteration of GOPC-ROS1 fusion and response to crizotinib. In this study, we reported a novel ROS1 fusion in breast cancer. CD74-ROS1 fusion transcripts have been detected in 0.7–1.9% of patients with NSCLC [11–14]. In studies of NSCLC patients, reverse transcription polymerase chain reaction (PT-PCR), fluorescence in situ hybridization (FISH), immunohistochemistry (IHC) and NGS were the most commonly used methods to confirm the fusion gene. However, there are no approved companion assays for the diagnosis of CD74-ROS1 fusion. In this study, NGS and IHC were used for the analysis of the CD74-ROS1 fusion gene in the patient with IBC. We confirmed the CD74-ROS1 fusion gene for the first time in an IBC patient by NGS. In the IHC analysis, overexpression of the ROS1 protein in breast tumor tissue was confirmed. This is in line with results from other studies which showed overexpression of the ROS1 protein in primary lung tumor tissue and lymph node metastases when the CD74-ROS1 fusion gene was present in these tissues [3]. Crizotinib, a small-molecule inhibitor against anaplastic lymphoma kinase (ALK) and ROS1 kinase, showed an objective response rate of 72% in a patient with NSCLC and ROS1 fusion [15]. For our patient harboring the CD74-ROS1 fusion gene, ROS1 fusion might be a viable drug target. Unfortunately, this IBC patient refused to undergo crizotinib treatment for financial reasons.

Other than the fusion gene, high mutation frequency of TP53BP1 and ARID1A was detected in this IBC patient. The TP53BP1 and ARID1A nonsense mutation frequency was 21% and 19.9%, respectively. As tumor suppressor genes, TP53BP1 and ARID1A have different roles in gene repair and stability. 53BP1 functions in two major double-strand break repair pathways, promoting nonhomologous end-joining (NHEJ) and inhibiting homology-directed repair (HDR), and integrates cellular input to ensure their timely execution in the proper cellular contexts [16]. ARID1A plays a role in targeting SWI/SNF complexes to tissue-specific enhancers and in maintaining their chromatin accessibility, controlling the cell cycle and DNA damage checkpoint, and regulating P53 and telomerase activation [17]. The CD74-ROS1 fusion gene, in conjunction with the TP53BP1 and ARID1A nonsense mutations, may have contributed to the rapid progression of IBC disease in this patient, with only 18 months from discovery of breast lump to death.

In conclusion, this is the first report of a CD74-ROS1 fusion gene in a patient with IBC. Although the effect of crizotinib in patients with IBC harboring the CD74-ROS1 fusion gene remains unclear, genetic sequencing may help us in designing individualized treatment.

## Data Availability

The datasets used and/or analyzed during the current study are available from the corresponding author on reasonable request.
